# Global research trends and hotspots for leukocyte cell-derived chemotaxin-2 from the past to 2023: a combined bibliometric review

**DOI:** 10.3389/fimmu.2024.1413466

**Published:** 2024-05-31

**Authors:** Wei Liu, Qin Wang, Jianishaya Yeerlan, Yirui Yan, Luke Xu, Cui Jia, Xinlian Liu, Lushun Zhang

**Affiliations:** ^1^ Department of Neurology, Nanbu People’s Hospital, Nanbu, China; ^2^ School of Laboratory Medicine, Chengdu Medical College, Chengdu, China; ^3^ School of Clinical Medicine, Chengdu Medical College, Chengdu, China; ^4^ Development and Regeneration Key Laboratory of Sichuan Province, Institute of Neuroscience, Department of Pathology and Pathophysiology, Chengdu Medical College, Chengdu, China

**Keywords:** LECT2, bibliometrics, CiteSpace, VOSviewer, visualization

## Abstract

Leukocyte cell-derived chemotaxin-2 (LECT2) is an important cytokine synthesized by liver. Significant research interest is stimulated by its crucial involvement in inflammatory response, immune regulation, disease occurrence and development. However, bibliometric study on LECT2 is lacking. In order to comprehend the function and operation of LECT2 in human illnesses, we examined pertinent studies on LECT2 investigation in the Web of Science database, followed by utilizing CiteSpace, VOSview, and Scimago Graphica for assessing the yearly quantity of papers, countries/regions involved, establishments, authors, publications, citations, and key terms. Then we summarized the current research hotspots in this field. Our study found that the literature related to LECT2 has a fluctuating upward trend. “Angiogenesis”, “ALECT2”, “diagnosis”, and “biliary atresia” are the current investigative frontiers. Our findings indicated that liver diseases (e.g. liver fibrosis and hepatic cell carcinoma), systemic inflammatory disease, and amyloidosis are the current research focus of LECT2. The current LECT2 research outcomes are not exceptional. We hope to promote the scientific research of LECT2 and exploit its potential for clinical diagnosis and treatment of related diseases through a comprehensive bibliometric review.

## 1 Introduction

Leukocyte-derived chemokines-2 (LECT2) is a 16 kDa chemokine that mediates neutrophil migration, and it belongs to the interleukin-8 family (1). The LECT2 gene is situated on chromosome 5q31.1-q32 (2). It is highly similar to the sequence of the chondromodulin repeat region of the chicken myb-induced myeloid 1 protein (3). This gene has the Val58Ile polymorphism regarding rheumatoid arthritis. Therefore, it is also called chondromodulin-II (ChM-II or CHM2) (4, 5). Human LECT2 protein contains three internal disulfide connections (Cys25-Cys60; Cys36-Cys41; Cys99-Cys142). Zinc binds to its disulfide bonds to inhibit the self-oligomerization of LECT2 *in vitro*, thereby stabilizing the LECT2 structure (6, 7).

LECT2 primarily produces by hepatocytes, released into the bloodstream (8). It is widely distributed in the liver, kidneys, intestines, skin, and brain (9–15). It is also expressed in many tissues, including muscle cells, endothelial cells, adipocytes, lymph nodes, spleen, bone marrow, and other immune tissues (16–19).

A series of research indicated that the LECT2 protein could play a role in the development and advancement of various conditions. Among immune system diseases like osteoarthritis (20) and rheumatoid arthritis (21), septicemia (22), atherosclerosis, osteoporosis (23), diabetes (18), atopic dermatitis (24), epithelial ovarian cancer (25), and obesity (26), LECT2 can activate and recruit immune cells to regulate inflammatory responses and immune responses (9). LECT2 regulates the bone microenvironment and promotes chondrocyte proliferation (20). New research has uncovered that in the pathogenesis of hepatocellular carcinoma (HCC), LECT2 can improve the tumor microenvironment (27, 28), and prevent vascular invasion and metastasis in HCC (29). Leukocyte chemotactic factor 2 amyloidosis (ALECT2) mainly expressed in the liver or kidney, and the overexpression of LECT2 is one of the important causes of ALECT2 amyloid deposition (30–32). Some researchers have found that LECT2 could promote the development of nerve cells in the brain (33, 34). Regarding obesity and insulin resistance, overexpression of LECT2 reduces insulin receptor substrate (IRS-1) levels (26, 35). The above studies have revealed the potential of LECT2 as a therapeutic target for related diseases. However, demand for more advanced research is critical because more complex mechanisms need to be further elucidated.

Currently, there is no bibliometric research on LECT2. Therefore, we utilize VOSviewer and CiteSpace bibliometric software for visual examination of the LECT2 literature from the Web of Science Core Collection (WoSCC). This method intertwines mathematical statistics, providing a systematic qualitative and quantitative evaluation. One of its benefits is the ability to assess the impact and contributions of various authors, countries, institutions, etc. general information. Additionally, it can illustrate the evolution of the field, identify research trends, and explore cutting-edge topics using the Scientific Knowledge Graph. This paper shows the macro development of LECT2 research, discusses the current research hotspots and frontiers, and provides a reference for follow-up related research.

## 2 Method

### 2.1 Search strategy and data collection

In order to guarantee the reliability and availability of the information, we conducted a search in the Web of Science Core Collection (WoSCC) database for articles on LECT2. The search term is “ TS= (“LECT2” OR “Leukocyte cell-derived chemotaxin-2” OR “Chondromodulin-II” OR “ChM-II” OR “CHM2”)”. According to the purpose and content of this study, the disciplines of the Web of Science classified as fisheries, veterinary science, marine freshwater biology, zoology, oceanography, remote sensing, agriculture, dairy, and animal science were excluded. The publication period is limited from January 1, 2010 to December 31, 2023, reasonably. For one thing, the earliest publication relevant to the topic of this review was published in 2010. For another thing, the number of publications published in 2024 is too small to have a profound impact. Since the total number of papers on LECT2 is not large, there is no restriction on the type of publications. To avoid bias caused by the daily update of the database, we collected the search data on January 27, 2024, and obtained a total of 181 articles, including article (n=125), meeting abstract (n=33), review article (n=12), editorial material (n=6), letter (n=3), correction (n=1), retraction (n=1). The record format is all records and references. The data is in plain text, and then imported into CiteSpace. These 181 articles obtain 3928 different references, with 2857 citations, an average of 15.78 times per item, and 30 h-indexes.

### 2.2 Data analysis

**Figure 1:**
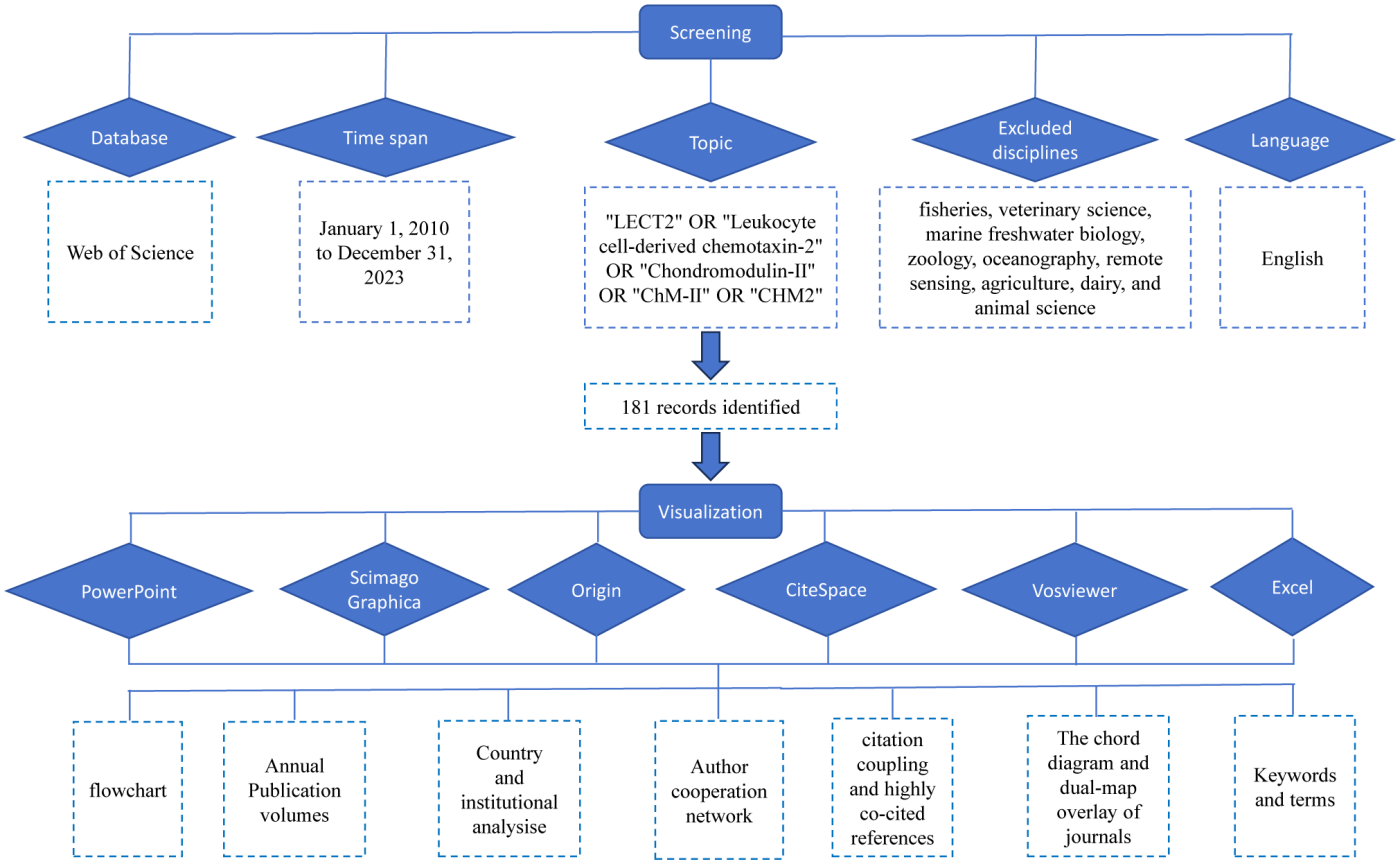
The flowchart illustrating the search strategy and selection process.

This study used Microsoft Office Excel 2021, VOSviewer (v1.6.20), CiteSpace (v6.2.R7), Origin, Pajek, and Scimago Graphica to analyze 181 collected documents. **Figure 1** is a flowchart of the data acquisition and visual analysis of this study. VOSviewer, a Java-based software created in 2009 by Van Eck and Waltman from the Center for Science and Technology Research (CWTS) at Leiden University in the Netherlands, is available for free. It has great features in displaying bibliometric maps, commonly used to build collaborative, co-quoting, and co-occurrence networks (36). The combination of VOSview with Scimago Graphica and Pajek makes images and data more diverse. CiteSpace, a program created by Professor Chaomei Chen from Drexel University in the US, is used for analyzing and visualizing bibliometric data. The advantage of this approach is that it shows the turning point of the scientific revolution through visually prominent nodes and connections to discover research hotspots and frontiers (37). According to the features and advantages of the above tools, we make use of them to conduct corresponding Scientific Knowledge Graphs.

## 3 Results

### 3.1 The annual number of publications and trends

**Figure 2:**
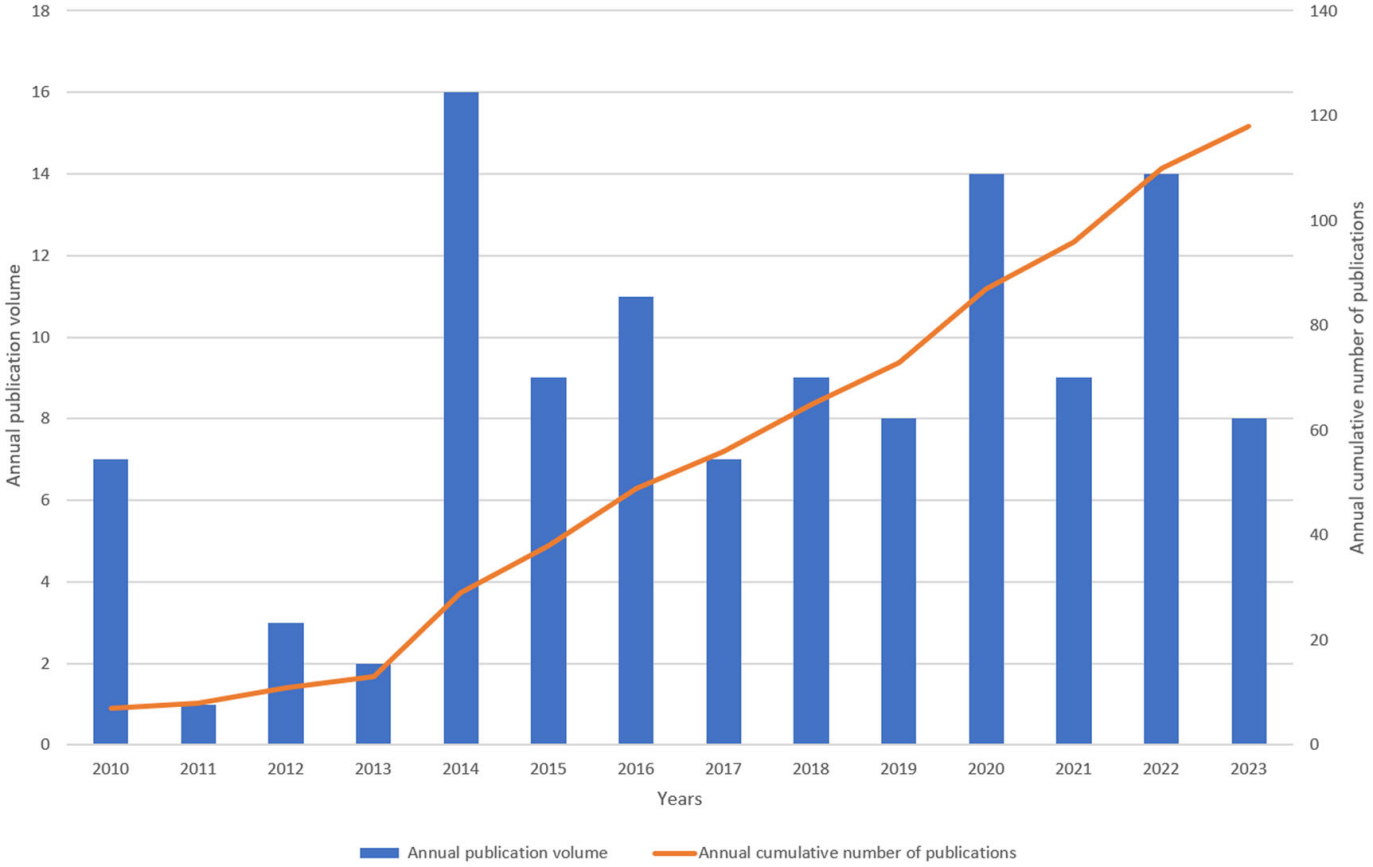
Annual output of research of leukocyte cel1-derived chemotaxin-2.

The annual number of publications can reflect the research process and trend in this field to a certain extent. In **Figure 2**, the increase in publications and citations is depicted. Between 2010 and 2021, there were notable fluctuations in the yearly numbers, with a general upward trend. The highest annual number of publications is 21 in 2020. The most significant increase is from 2013 to 2014, reaching 13 publications, suggesting a major advancement in LECT2 research during that period. The large number of publications in the past two years indicates that this field has attracted people’s attention and has a good development trend. It is now garnering increasing interest from scholars.

### 3.2 Country/region and institutional analysis

There are 32 countries/regions and 237 institutions in total. The rankings, quantities, proportions, citation frequencies, and centralities of the top ten countries in terms of publications are displayed in **Table 1**. The top three countries/regions for publication are the United States (n = 66), mainland China (38), and Japan (39). The top three countries/regions in centrality are the United States (1.08), Germany (0.25), and mainland China (0.19), indicating that their research results have a greater impact on LECT2 research.

**Table 1:** Top 10 countries on research of LECT2.

Rank	Countries/Regions	Publications	Citations	Centrality
1	USA	66(36.5%)	1343	1.08
2	China Mainland	52(28.7%)	658	0.19
3	Japan	40(22.1%)	692	0.02
4	England	12(6.6%)	161	0.14
5	South Korea	9(5.0%)	204	0.02
6	Taiwan, China	7(3.9%)	96	0
7	Canada	6(3.3%)	81	0
8	France	5(2.8%)	307	0.01
9	Germany	5(2.8%)	167	0.25
10	Mexico	5(2.8%)	15	0

**Figure 3:**
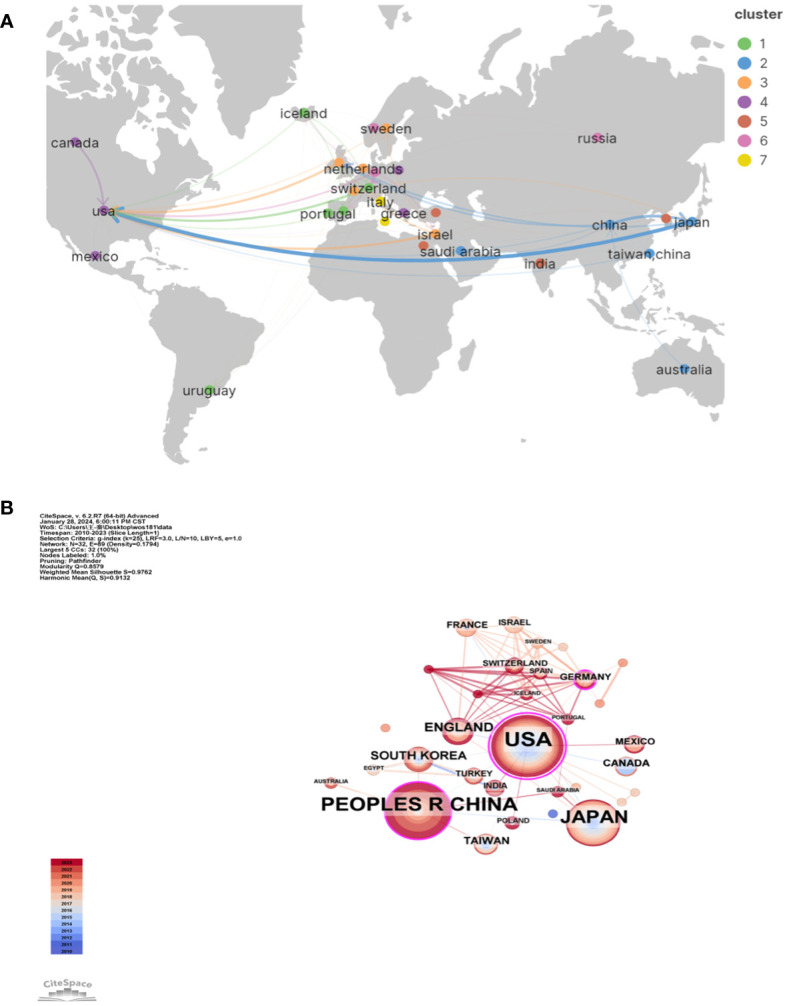
The geographical distribution **(A)** and visualization of countries **(B)** on research of leukocyte cell-derived chemotaxin-2.

We employed VOSviewer and Scimago Graphica to generate a national geographic visualization atlas (**Figure 3A**). CiteSpace was used to generated a collaborative network analysis map amongst 32 countries, visualizing publications and partnerships. Labels denote nations with more than two publications (**Figure 3B**). Node sizes represent the number of publications; link width denotes partnership strength. As evident from **Figures 3A, B**, the USA leads in transnational cooperation, partnering with 21 nations including Italy, India, Poland, China, and so on. In Europe, Iceland, Netherlands, Switzerland, Portugal have carried out extensive and close cooperation on LECT2-related research.

**Figure 4:**
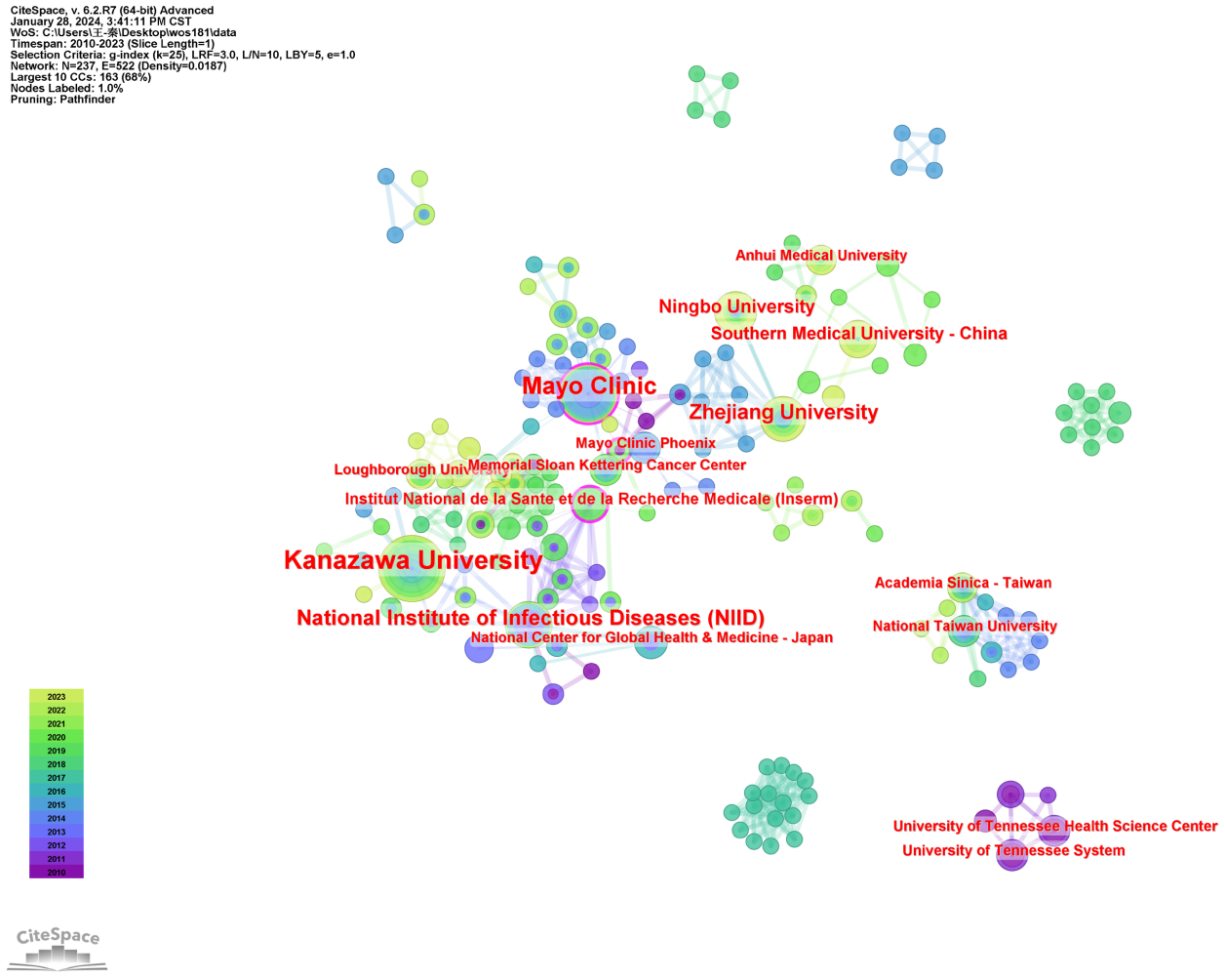
The visualization of institutions on research of leukocyte cell-derived chemotaxin-2.

Using publication volumes as an indicator, each institute’s research output on LECT2 research is counted. The top 10 institutions on research of LECT2 are displayed in **Table 2**. Kanazawa University had the most publications with 19 articles, while the Mayo Clinic and the National Institute of Infectious Diseases followed with 15 and 10 articles respectively. The institutional collaboration map was created filtering institutions publishing less than ten times. As shown in **Figure 4**, institutional cooperation is relatively close. Mayo Clinic collaborated with 25 organizations such as Mayo Clinic Phoenix and Memorial Sloan Kettering Cancer Center. The National Institute of Health and Medical Research also maintains numerous collaborations with various institutions.

**Table 2:** Top 10 institutions on research of LECT2.

Rank	Institution	Counts	Citations	Centrality
1	Kanazawa University	19(10.5%)	246	0.05
2	Mayo Clinic	15 (8.3%)	426	0.16
3	National Institute of Infectious Diseases (NIID)	10 (5.5%)	380	0.09
4	Zhejiang University	9 (5.0%)	187	0.05
5	Ningbo University	7 (3.9%)	143	0
6	Southern Medical University - China	6 (3.3%)	34	0
7	Institut National de la Sante et de la Recherche Medicale (Inserm)	5(2.8%)	307	0.15
8	Academia Sinica - Taiwan	4(2.2%)	83	0
9	Anhui Medical University	4(2.2%)	116	0
10	Loughborough University	4(2.2%)	90	0

### 3.3 Analysis of authors partnerships

**Table 3** lists the top 10 authors with the most posts. Of these authors, Satoshi Yamagoe has been cited the most. Toshinari Takamura has the highest number of articles with 14, followed by Hirofumi Misu with 13, and Shuichi Kaneko with 11. All of them are from Kanazawa University, and their research on LECT2 is biased toward the association of LECT2 with obesity and insulin resistance. The concept of co-citation is a research method to measure the degree of relationship between documents. **Table 3** also displays the top ten authors who have the highest number of co-citations in academic works. The top three co-citations are Yamagoe S (159 articles), Larsen CP (78 articles), and Okumura A (74 articles).

**Table 3:** Top 10 authors and co-cited authors on research of LECT2.

Rank	Authors	Documents	Citations	Co-Cited Authors	Citations
1	Toshinari Takamura	14	118	Satoshi Yamagoe	159
2	Hirofumi Misu	13	89	Christopher P Larsen	78
3	Shuichi Kaneko	11	55	Akihisa Okumura	74
4	Satoshi Yamagoe	10	308	Xin-Jiang Lu	66
5	Takayoshi Shirasaki	8	0	Merrill D Benson	65
6	Tetsuro Shimakami	8	0	Fei Lan	55
7	Kazuhisa Murai	8	0	Samar M Said	44
8	Masao Honda	8	0	Chi-Kuan Chen	40
9	Hiroaki Takayama	7	118	Takafumi Saito	39
10	Jiong Chen	7	143	Hwan-Jin Hwang	38

**Figure 5:**
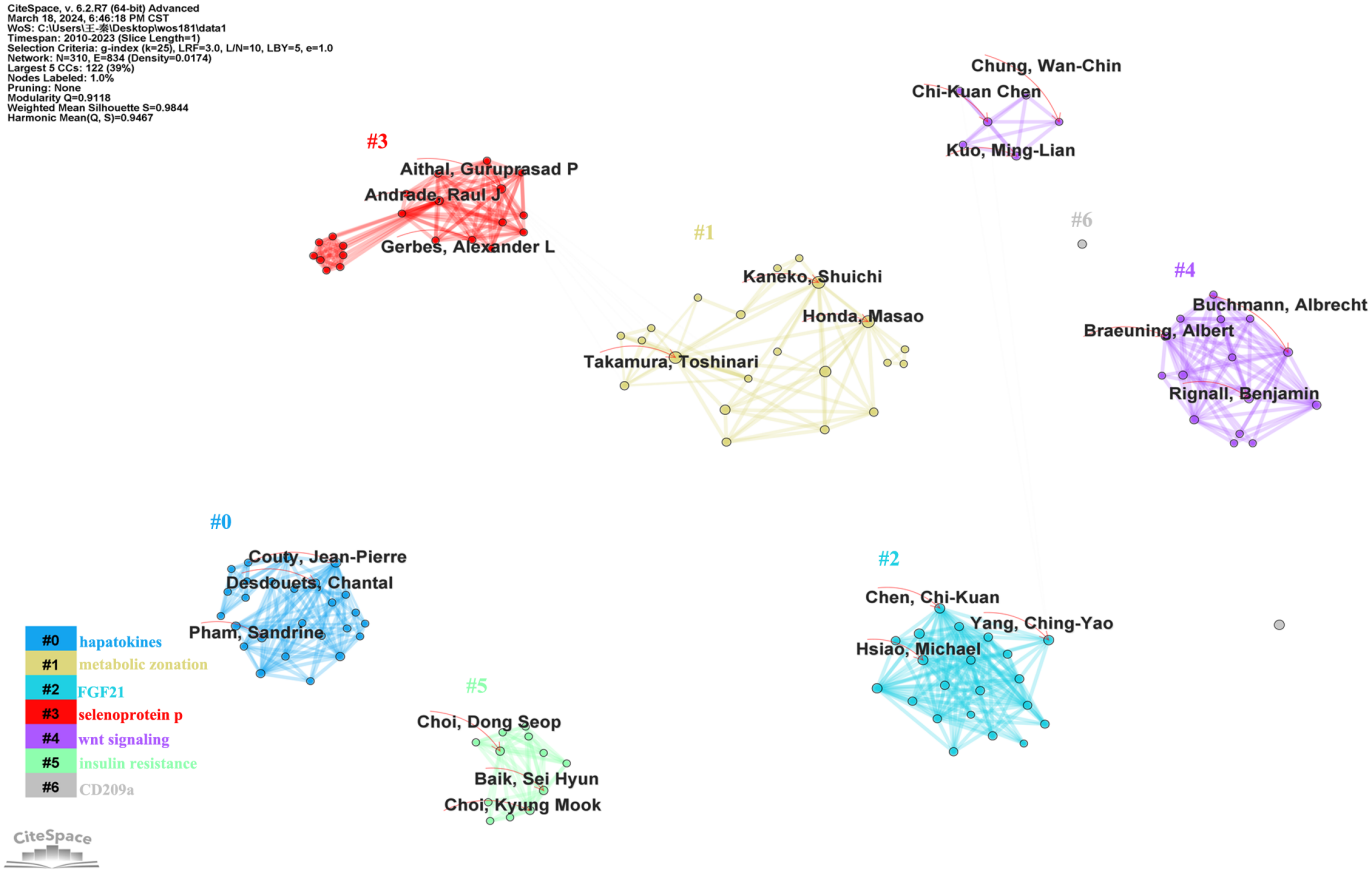
The author cooperation network on research of leukocyte cell-derived chemotaxin-2.

A total of 1005 authors were included in the literature data. The author cooperation network generated by CiteSpace was used to show the collaboration of 310 authors. The lines linking the nodes represent the cooperative relationships between authors, and the sizes of the nodes indicate the quantity of articles published on LECT2 by each author. Based on keywords, the authors’ collaborative research was divided into 7 clusters, labeled #0- #6, and corresponding to the keywords shown in the color bars in **Figure 5**. Nodes of the same cluster are filled with the same color. In terms of Sigma, a metric that measures importance, it can be concluded that the top ranked author Aithal, Guruprasad P made a very important contribution to the research in this field. According to the lines in the diagram to analyze the collaboration between the authors, Takamura Toshinari and Shuichi Kaneko were the most active collaborators (**Figure 5**). Satoshi Yamagoe, Toshinari Takamura, and Shuichi Kaneko collaborated to uncover the therapeutic potential of LECT2 in treating infectious diseases and cancer (40). The cooperation between the authors promotes the breadth and depth of LECT2 research directions.

### 3.4 Analysis of journals and co-cited journals

A total of 118 journals published articles on LECT2. HEPATOLOGY had the highest number of publications with 17, while *PLOS ONE* came in second with 6. *KIDNEY INTERNATIONAL* ranks first in impact factor among the top 10 journals according to **Table 4**.

**Table 4:** Top 10 journals on research of LECT2.

Rank	Journal	Record Count	IF(2022)	JCR
1	HEPATOLOGY	17	3.6	Q2
2	PLOS ONE	6	3.7	Q2
3	AMYLOID-JOURNAL OF PROTEIN FOLDING DISORDERS	5	5.5	Q2
4	RONTIERS IN IMMUNOLOGY	5	7.3	Q1
5	KIDNEY INTERNATIONAL	5	19.6	Q1
6	AMERICAN JOURNAL OF KIDNEY DISEASES	4	13.2	Q1
7	APPLIED PHYSIOLOGY NUTRITION AND METABOLISM	3	3.4	Q2
8	FRONTIERS IN GENETICS	3	3.7	Q2
9	JOURNAL OF BIOL0GICAL CHEMISTRY	3	4.8	Q2
10	MODERN PATHOLOGY	3	7.5	Q1

**Figure 6:**
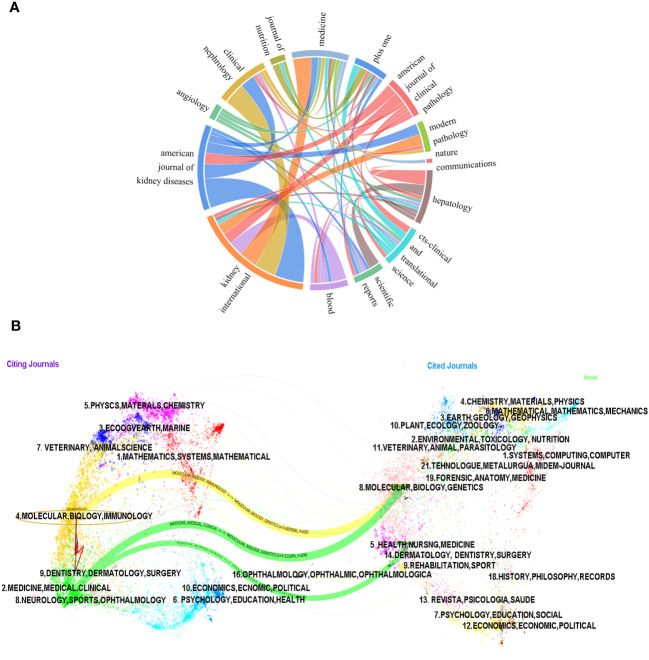
The chord diagram **(A)** and the dualmap overlay of journals **(B)** on research of leukocyte cell-derived chemotaxin-2 to illustrate journal citation connections.

We employed a chord diagram to illustrate journal citation connections (**Figure 6A**). Each colorful track signifies a journal, positioned radially. The string thickness visually quantifies the correlation strength. *KIDNEY INTERNATIONAL* boasts an extensive correlation with *AMERICAN JOURNAL OF KIDNEY DISEASES*, and a a relatively strong one with *CLINICAL NEPHROLOGY*. It can be concluded that the citation connections between journals are highly active.

The dual-map overlay of journals illustrates the citation connections among the leading edge of knowledge and the knowledge base in the macrostructure. There are four macro structural development models: independence, divergence, convergence, and intersection. Understanding them can help researchers screen important references and grasp the historical development path of the topic. In **Figure 6B**, on the left is the distribution of journals where the original documents are located; on the right is the distribution of journals where the corresponding cited literature is located. The width of the link reflects how often it is referenced. The graph displays the top three significant pathways. The results of the double graph superposition show that hot journals (such as *HEPATOLOGY*) are mainly concentrated in the fields of molecular biology and immunology (see the center of the circle on the left). Hot cited journals are mainly concentrated in the fields of molecular biology, genetics (such as *HEPATOLOGY*, *BLOOD*) and health, nursing, medicine (such as *KIDNEY INT*) (see the center of the circle on the right).

The results also confirmed that the research hotspots of LECT2 have evolved from molecular biology and gene fields to immunology and medicine, medical, clinical respectively, while the research in the fields of health, nursing, and medicine has evolved to medicine, medical, and clinical.

### 3.5 Analysis of citation coupling and highly co-cited references

**Figure 7:**
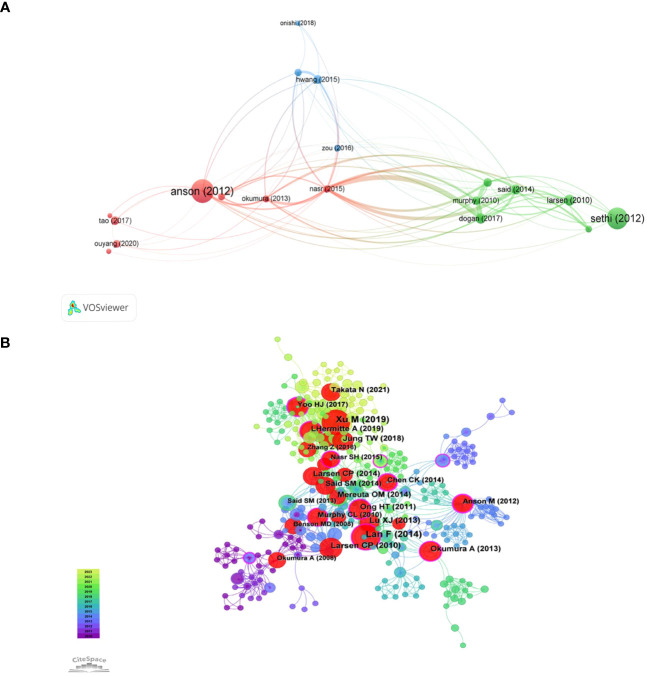
The visualization of f citation coupling **(A)** and and highly co-cited references **(B)** on research of leukocyte cell-derived chemotaxin-2.

Citation coupling is a method to study the internal connection of scientific literature. If both papers cite the same article, then both citations will be considered a citation pair. The degree of coupling reflects the degree of similarity to the literature research topic. In finding relevant literature and determining the direction of research, scholars are given better advice. VOSviewer was used to perform citation coupling analysis on the top 20 most cited articles (with a minimum of 33 citations). Nineteen of them form a total of 91 coupling relationships (**Figure 7A**). Each node represents a document, marked with the name of the first author and the year of publication. The four nodes marked as Nasr (2015), Dogan (2017), Said (2014), and Murphy (2010) are very tightly connected, indicating that the four articles are highly correlated with each other.

When multiple papers are referenced by another paper simultaneously, it forms a co-citation connection. Co-citation analysis is an evolving framework that can assist researchers in comprehending the latest advancements in the field of study. The **Supplementary Materials** show the top ten co-cites in the literature. The article titled “LECT2, a Ligand for Tie1, Plays a Crucial Role in Liver Fibrogenesis” is the most frequently cited. We use CiteSpace to generate a co-citation network of 413 nodes and 1523 connections, with a density of 0.0179 (**Figure 7B**). Overall, the co-occurrence relationship between pieces of literature is strong, and multiple nodes act as bridges between subgroups. The 12 purple outer ring nodes are the literatures with high intermediary centrality (Centrality greater than 0.1), which explains the significance. Further analysis of the main content of the literature, there are 5 articles related to liver diseases, 4 articles related to inflammatory immune mechanisms, and 3 articles related to obesity and amyloid lesions.

Through the above two analysis methods, representative documents can be screened by combining the frequencies of citations, the frequencies of co-citations, and the centrality of literature. In the article titled “LECT2 Controls Inflammatory Monocytes to Constrain Growth and Progression of Hepatocellular Carcinoma”, the authors analyze how LECT2 influences the progression of HCC and suggest that LECT2 could be a beneficial immunotherapy choice for HCC. Another study revealed that LECT2 acts as a hepatokine connecting obesity with insulin resistance in skeletal muscle. It was discovered that the LECT2 protein hindered insulin signaling by phosphorylating Jun NH2-terminal kinase in C2C12 myocytes.

### 3.6 Analysis of keywords and burst terms

**Figure 8:**
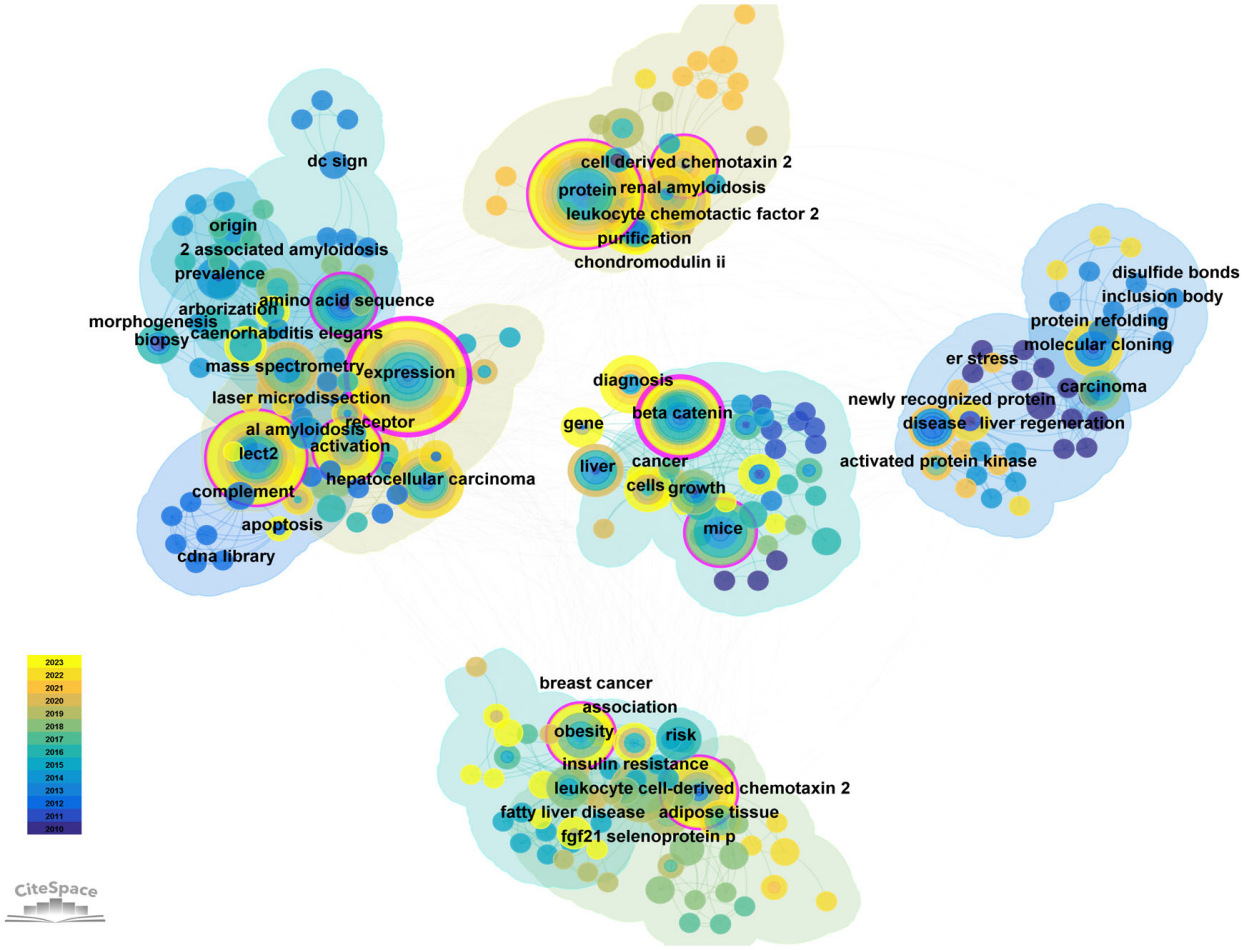
The cluster of keywords on research of LECT2.

Keyword cooccurrence analysis reveals research hotspots by analyzing high-frequency keywords and their correlations. **Table 5** lists the keywords, centrality, and first appearance for the top 20 cooccurrences. Intermediary centrality analysis reveals the mutation or transformation of research hotspots. CiteSpace was used, resulting in 294 keywords. Larger nodes correspond to more frequent cooccurrences. Purple outer ring node is an important keyword: expression, amino acid sequence, activation, beta-catenin, protein, obesity (**Figure 8**).

**Table 5:** Top 20 keywords on research of LECT2.

Rank	keywords	Freq	Centrality	Year
1	expression	39	0.33	2010
2	protein	34	0.17	2010
3	beta catenin	15	0.21	2011
4	leukocyte cell-derived chemotaxin 2	14	0.14	2010
5	hepatocellular carcinoma	14	0.08	2013
6	activation	12	0.11	2012
7	amino acid sequence	12	0.17	2010
8	obesity	11	0.13	2015
9	insulin resistance	11	0.07	2017
10	purification	10	0.03	2010
11	diagnosis	10	0.07	2014
12	molecular cloning	9	0.09	2010
13	liver	9	0.04	2010
14	mass spectrometry	9	0.08	2013
15	renal amyloidosis	9	0.04	2015
16	chondromodulin ii	6	0.01	2013
17	disease	6	0.09	2010
18	selenoprotein p	5	0.01	2018
19	fatty liver disease	5	0.04	2010
20	biopsy	5	0.02	2015

In the timeline (**Supplementary Figure 1**), each line is a cluster, numbered #0 #1 #2 #3, etc. and a total of 8 clusters are formed. The cluster reflects the temporal characteristics of the research field. The number of keywords in cluster #0 #1 #2 #3 #4 are the largest. The first group of human hepatocellular carcinoma (cluster #0) contains the keywords beta-catenin, gene expression, and diagnosis. etc. This indicates that hepatocellular carcinoma may be related to LECT2. The second group is systemic inflammation (cluster #1). Its keywords are expression, hepatocellular carcinoma, chondromodulin II. This group focused on the association of leukocyte-derived chemotaxin 2 expressions with systemic inflammation or immune regulation. The third group is chemotactic factor (cluster #2). The 4th group (cluster #3) is JNK-dependent inhibition. The three most cited keywords are obesity, fatty liver disease, risk. This group of studies revealed the chemotactic effect of LECT2 on leukocytes. The 5th group (cluster #4) is selenoprotein p. Its research is linked to insulin resistance and green tea extracts. The keywords in the first five clusters indicate that LECT2 research has achieved more significant results in human hepatocellular carcinoma, systemic inflammation, chemotactic factor, selenoprotein p and JNK-dependent inhibition.

CiteSpace software can extract and detect burst terms to understand the research frontier, changes in research focus, and the latest research hotspot trends, and help predict future trends in the field. As shown in **Supplementary Figure 2**, 25 emergent words are obtained, showing the year of the initial appearance of the keyword, while Begin and End signify the beginning and conclusion of its relevance as a cutting-edge concept, with Strength denoting its emerging power. The red line indicates the particular period in history when the term gained popularity in academic research. Nodes that have not yet appeared are shown in a light blue color, while nodes that have started to appear are shown in a dark blue color. It is worth noting that insulin resistance (5.5) has the greatest emergent strength, followed by cell-derived chemotaxin 2 (2.7), selenoprotein p (2.67), and risk (2.56). From the time of appearance, diseases, and newly recognized proteins first appeared. Current research on LECT2 is focused on cell-derived chemotaxin 2, angiogenesis, leukocyte chemotactic factor 2 amyloidosis (ALECT2), diagnosis, and biliary atresia (BA), marking an emerging period of study.

## 4 Discussion

### 4.1 General information

Our bibliometric research assess the impact and contributions of various countries, institutions, authors, journals. The number of published articles exploded from 2013 to 2014, indicating a surge in interest among scholars. The countries with the most published papers are the United States, China, and Japan, demonstrating their maturity in this area. 118 journals have published research literature related to LECT, with *Hepatology* being the most prolific and *PLOS ONE* closely following. These journal data can be sued as a navigation for scholars aiming to publish in specific journals. Toshinari Takamura, Hirofumi Misu, and Shuichi Kaneko are the top three authors. The most cited author is Satoshi Yamagoe (n = 159) of the National Institute of Infectious Diseases (NIID). He and his research team firstly reported LECT2 protein in 1998, which is mainly produced by hepatocytes (2). Satoshi Yamagoe, Toshinari Takamura, and Shuichi Kaneko discovered that LECT2 interacts with MET (Mesenchymal to epithelial transition factor) to promote RIG-L-mediated innate immune responses. The findings hold promise for therapeutic applications in several infectious diseases and cancers (41).

### 4.2 Knowledge base

According to the result of co-citation network analysis in the literature, we can roughly determine the research basis of LECT2. In these 10 co-cited papers, the first, eighth, and tenth were on liver fibrosis and liver cancer; the second, sixth, and seventh were on obesity and insulin resistance; and the third, fifth, and ninth were on amyloidosis. Meng Xu et al. published the most co-cited study in *Cell* in 2019 (42). They found that LECT2 promoted the transformation of Tie1/Tie2 heterodimer to Tie2/Tie2. Tie1 and Tie2, two types of receptor tyrosine kinase (RTKs) on the surface of hepatic sinusoidal endothelial cells, are members of the TIE (Tyrosine kinase with immunoglobulin-like and EGF-like domains 1) receptor family that regulates angiogenesis. Overexpression of LECT2 leads to the deterioration of liver fibrosis by inhibiting portal vein angiogenesis and promoting sinus capillary formation (42). Additionally, Antoine L’Hermitte et al. and H T Ong all discovered the ability of LECT2 to inhibit the migration and growth of liver cancer cells (29, 39). Fei Lan, Okumura A and Tae Woo Jung proposed that LECT2 is a metabolism-related hepatokine, and predicted it as a therapeutic target for insulin resistance, respectively. It exerts a positive influence on Jun NH_2_-terminal kinase (JNK) stavation-sensing kinase adenosine monophosphate-activated protein kinase negatively regulates its expression (26). Tae Woo Jung found that LECT2 enhanced inflammation by acting on downstream targets such as P38 and CD209a. It also promotes lipid synthesis by regulating sterol regulatory element-binding protein 1c (SREBP1c) (35). Christopher P Larsen published 2 papers in these 10 total cited papers. He first discovered an amyloid deposit composed of LECT2 (ALECT2). It is considered to be the third form of renal amyloidosis (31, 43). Based on that basis, Oana M Mereuta typed the liver amyloid deposits (44). Their findings lay the foundation for further research into the effects of LECT2 on amyloidosis. Researchers from the Laboratory of Biochemistry and Molecular Biology, Ningbo University found that LECT2 specifically binds to the CD209a receptor and activates macrophages (22).

### 4.3 Research hotspots and frontiers

**Figure 9:**
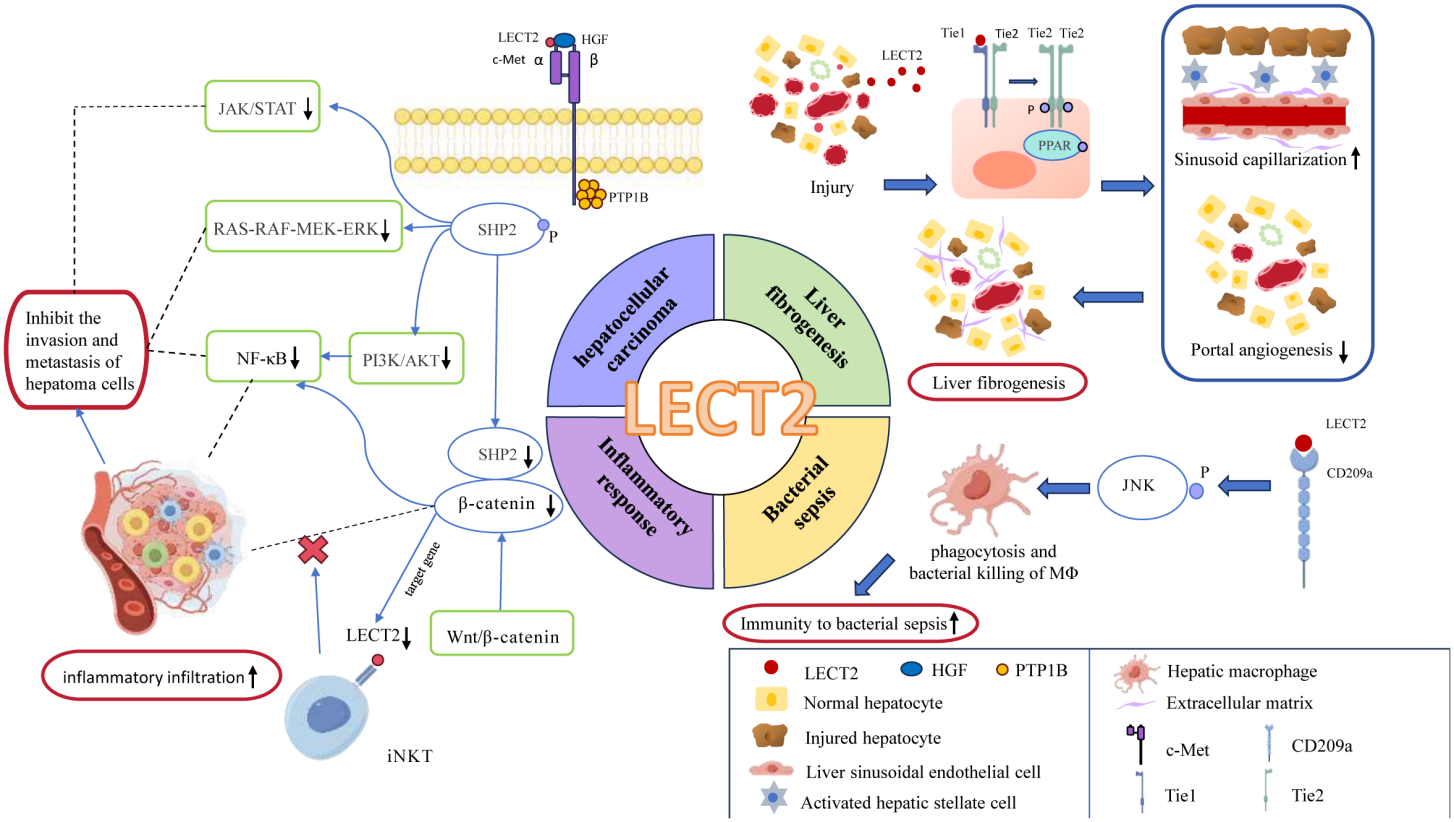
the mechanism diagram of LECT2’s involvement in the regulation of related diseases, including hepatocellular carcinoma, liver fibrogenesis, bacterial sepsis, inflammatory response. In this figure, blue ovals represent enzymes or proteins; red rounded rectangles represent effects; green rounded rectangles represent the signaling pathways; Tie1, Tie2: as tyrosine kinase receptors, they are subtypes of Tie; PPAR, peroxisome proliferators-activated receptors; JNK, c-Jun N-terminal kinase; c-Met, cellular-mesenchymal epithelial transition factor; HGF, hepatocyte growth factor; PTP1B, Protein Tyrosine Phosphatase-1B; SHP2, Src homology 2 domain-containing protein tyrosine phosphatase.

Our methodological review focuses on the top 25 terms with the strongest citation bursts to identify the frontiers of LECT2, including Angiogenesis, ALECT2, cell-derived chemotaxin 2, diagnosis, and BA (Biliary Atresia). Angiogenesis affects the pathological stages of liver fibrosis and hepatocarcinogenesis, associated with RA, inflammation (45). Therefore, we can predict the pathway in which LECT2 affects these diseases. ALECT2 is an amyloidosis that occurs frequently in the kidney and liver. It is commonly seen in patients with nephrotic syndrome and azotemia triggered by overexpression of LECT2 (46). Cell-derived chemotaxin 2 is the third research hotspot, indicating that LECT2 mainly plays the biological function of chemotactic centrocytes in immune response. The diagnosis of diseases is also a hot research direction of LECT2, especially for tumors like HCC and breast cancer (13, 40, 47, 48). BA is the fifth research hotspot to study the mechanism of LECT2-induced liver fibrosis in patients with Biliary Atresia (49). We drew a mechanism diagram by Figdraw to illustrate how LECT2 affects the development of related diseases, including HCC, liver fibrogenesis, bacterial sepsis, inflammatory response (**Figure 9**). According to disease classification and the specific mechanism, the current research hotspots of LECT2 will be elucidated in each of the following sections.

#### 4.3.1 Association of LECT2 with systemic inflammatory disease

Systemic inflammation is one of the research hotspots of LECT2. LECT2 has neutrophil chemotaxis and is involved in inflammatory and immune regulation processes. In general, LECT2 has immune cell activation, inflammatory mediator regulation, and anti-inflammatory effects (50, 51). Systemic inflammatory diseases related to LECT2 mainly include bacterial sepsis (22), atherosclerosis (52), osteoporosis (23), arthritis (38), and atopic dermatitis (24).

##### 4.3.1.1 Anti-inflammatory effect of LECT2 in bacterial sepsis

LECT2 can chemotaxis immune cells and pathogenic phagocytose microorganisms, but the exact mechanism by which LECT2 exerts its anti-inflammatory effects is unclear. A large number of research results can provide some clues. LECT2 can directly interact with CD209a receptors to boost macrophages’ capacity to phagocytose and eliminate bacteria through JNK phosphorylation (53). Then hepatic macrophages exert phagocytosis and bactericidal effects, thereby improving the body’s immunity to bacterial sepsis (22). This finding provides a basis for LECT2 as a new targeted therapy for sepsis. Furthermore, LECT2 can also reduce tumor necrosis factor (TNF) expression via CD209a receptor and maintain HSC (hematopoietic stem cells) homeostasis (54). These findings may help in the treatment of various blood diseases.

##### 4.3.1.2 Atherosclerosis

AS is a chronic immune inflammatory disease. The development of this condition involves various factors like dysfunctional endothelial cells, heightened levels of vascular adhesion molecules, elevated production of inflammatory cytokines, and accumulation of cholesterol in the walls of blood vessels. LECT2 may participate in these pathological processes, thus affecting the development of atherosclerotic lesions (52). Fatma Cavide Sonmez et al. proposed that LECT2 could be used as a new marker of AS (55). They found that LECT2 in atherosclerotic areas tended to be overexpressed through aortic wall perforation biopsy and immunohistochemical staining, which was confirmed in later studies (17, 56). Additional research has indicated that increased levels of LECT2 can impede the progression of AS. A genetically modified animal model containing the LECT2 gene is utilized to evaluate metabolic factors associated with the development of AS, including reduced levels of total cholesterol and low-density lipoprotein in the blood; lower levels of inflammatory cytokines and mRNA for monocyte chemoattractant protein-1 (MCP-1), matrix metallopeptidase 1 (MMP-1), tumor necrosis factor-α (TNF-α), interleukin-1β (IL-1β), and interleukin-8 (IL-8); higher quantities of smooth muscle cells and fewer CD68 macrophages, while CD31 endothelial cells remain unchanged (17). In contrast, another study found that the levels of intercellular adhesion molecule-1 and TNF-α, MCP-1, and IL-1β are elevated in LECT2-treated human umbilical vein endothelial cells and Tohoku Hospital Pediatrics-1 (THP-1 cells). These results indicated that LECT2 promotes the inflammatory response in AS. Therefore, the role of LECT2 in AS is controversial and needs to be confirmed by further researches (53).

##### 4.3.1.3 Rheumatoid arthritis, osteoarthritis, and osteoporosis

As a chronic systemic disease, the key pathophysiological process of RA is the progressive destruction of articular cartilage and bone. This process also includes the imbalance of osteoclasts, mesenchymal stem cells, osteoblasts, and endothelial cells within the bone environment, as well as irregular chemotaxis and infiltration of inflammatory cells (20).

The occurrence of RA and osteoarthritis is connected to the G/A polymorphism at nucleotide 172 in exon 3 of the ChM-II gene (21). Research has analyzed the genetic code and X-ray injuries of individuals with RA and discovered that having the 172A gene variant notably raises the risk of developing RA and the severity of joint deterioration (38). In line with this finding, the arthritis severity was notably higher in LECT2-deficient mice compared to the wild-type control group in the collagen antibody-induced arthritis (CAIA model) induced by anti-type II collagen antibody. The distinct signs included damage to cartilage and bone, increased inflammation, and elevated levels of proinflammatory cytokines like IL-1β and IL-6 (57). In subsequent studies, the exogenous expression of LECT2 reduced the RA phenotype of LECT2 (-/-) mice (20).

Collectively, the aforementioned researches suggested that LECT2 acts as a suppressor in regulating RA. The precise molecular mechanism by which LECT2 regulates RA remains unknown. Further laboratory experiments are needed to investigate how LECT2 influences arthritis-related cytokines.

The development of osteoporosis could also be linked to LECT2. Recent research indicated that there was a notable increase in serum LECT2 levels in individuals with osteoporosis, which impacted the severity of osteopenia (23). Certain academics forecast that serum LECT2 could serve as a promising indicator for evaluating the likelihood of bone deterioration (58).

Research on RA, osteoarthritis, and osteoporosis suggests that LECT2 plays a role in controlling bone immune responses, making it crucial for bone health.

#### 4.3.2 Association of LECT2 with liver disease

Numerous studies have demonstrated the involvement of LECT2 in various liver conditions, including hepatitis, acute liver failure (50), liver fibrosis (59), nonalcoholic fatty liver disease (60), cirrhosis, and HCC (29).

##### 4.3.2.1 Liver injury and hepatitis

Previous research has shown that removing LECT2 during concanavalin A (ConA) -induced hepatitis can disturb the balance of Natural killer T cell (NKT cells) in the liver. In LECT2 (-/-) mice, NKT cells are significantly increased. Elevated levels of IL-4, Interferon-gamma (IFN-γ), and cytotoxicity towards syngeneic thymocytes are observed as well (61). Nevertheless, the quantity of traditional T cells, natural killer cells, and various other cell varieties stays constant according to a study (50). Additional research has uncovered how LECT2 controls the progression of hepatitis. LECT2 has the ability to trigger the signaling pathways of TGF-β-activated kinase 1 (TAK1), Mitogen-activated protein (MAP) kinase kinase 4 (MKK4), and JNK, converting remaining liver macrophages into an m1-like state and advancing liver inflammation (62).

Yuan Xie, Ke-Bo Zhong et al. found that the function of LECT2 in the process of acute liver damage and recovery (50). They simulated liver damage caused by toxins and autoimmune reactions using carbon tetrachloride and concanavalin A (ConA) respectively. The degree of liver injury was evaluated by serum levels of Alanine Transaminase (ALT), Aspartate Aminotransferase (AST), and total bilirubin (Tbil). The results showed that LECT2 mRNA and serum LECT2 increased during the first to second days of exacerbation of liver injury. Compared with WT mice, LECT2-ko mice had less liver injury and less macrophage infiltration. These results confirmed the findings of Rachel J Church et al. (63).

Several research studies have indicated that LECT2 played a part in controlling acute liver damage and healing by managing the entry of various immune cells (64), and it could also serve as a potential biomarker for diagnosing liver injuries in clinical settings (10, 63, 65).

##### 4.3.2.2 Liver fibrogenesis

LECT2 has the effect of promoting liver fibrosis. The use of ICG-001 and LF3 to block β-catenin/TCF4 transcriptional activity resulted in decreased levels of LECT2, pSer 675 β-catenin, and nuclear β-catenin. The results showed that LECT2 was regulated by β-catenin/TCF4 signaling, thereby regulating angiogenesis and participating in liver fibrosis (59). LECT2 can bind to Tie1 and activate peroxisome proliferators-activated receptors (PPAR) signaling to transform Tie1/Tie2 heterodimers into Tie2-Tie2 homodimers, thereby inhibiting endothelial cell invasion and metastasis. Knockdown of the LECT2 gene significantly promotes portal vein angiogenesis and sinusoidal capillary reduction, which alleviates the symptoms of liver fibrosis (42). Moreover, LECT2 is also considered a pivotal gene in BA liver fibrosis to boost fibrosis by triggering fibrous genes such as α-smooth muscle actin and collagen type I alpha 1 Chain (66). The above studies indicated that LECT2 could potentially be used as a biomarker to predict liver fibrogenesis. The use of AAV9-LECT2-shRNA combined with bevacizumab has shown promising outcomes in treating liver fibrosis during the development of new drugs (67).

##### 4.3.2.3 Nonalcoholic fatty liver disease

NAFLD is not a typical systemic inflammatory disease (68). However, inflammation plays a pivotal role in its progression, from hepatic steatosis to nonalcoholic steatohepatitis and cirrhosis (69). LECT2 levels significantly increased in NAFLD (26, 35, 70). Studies have found two ways in which LECT2 promotes liver fat accumulation, including the STAT-1 pathway and transforming residual hepatic macrophages into an m1-like phenotype (71). Hwan-Jin Hwang and Tae Woo Jung have discovered that in the development of hepatic steatosis, adenosine 5’-monophosphate (AMP)-activated protein kinase (AMPK) and JNK mechanisms could play a role in regulating LECT2 expression and inducing liver inflammation (72). Meanwhile, LECT2 can stimulate LPS-triggered MKK4 and TGF-beta activated kinase 1 (MAP3K7) binding protein 2 (TAB2), activating JNK pathway (16). Therefore, LECT2 has the function of promotion on NAFLD progression.

##### 4.3.2.4 Hepatocellular carcinoma

HCC is the first focus of LECT2 research at present. LECT2 affects the evolution of liver cancer by altering the tumor phenotype and tumor microenvironment (73). It effectively suppresses endothelial cell proliferation, migration, and angiogenesis (15, 74).

L’Hermitte A et al. found that the absence of LECT2 leads to an increase in immune infiltrations and promotes the epithelial-mesenchymal transformation of Ctnnb-1 mutant tumor hepatocytes (29). Other research findings indicate that LECT2 expression appears to contradict angiogenesis in HCC patients (39, 75). The growth and spread of HCC depend on angiogenesis (76). So individuals with elevated LECT2 levels tend to experience decreased vascular invasion and prolonged survival in cases of HCC (15). Prior research has revealed how LECT2 hinders the process of angiogenesis. LECT2 directly binds to vascular endothelial growth factor receptor 2 (VEGFR2), resulting in the inhibition of VEGF165-induced tyrosine phosphorylation of VEGFR2 and subsequent extracellular signal-regulated kinase and serine/threonine kinase AKT (also known as protein kinase B or PKB) phosphorylation (77). This provides insights into LECT2’s anti-tumor activity.

LECT2 can also directly bind to the MET receptor on liver tumor cells at aa 159–175 of the α chain, preventing the interaction between the tyrosine kinase receptor MET and its ligand hepatocyte growth factor (HGF). Suppressing the downstream pathway of the HGF/MET axis, including phosphoinositide 3-kinase (PI3K)/AKT, RAS-RAF-MEK-ERK, and JAK/STAT, hinders the invasion and metastasis of hepatoma cells (74). Interaction between LECT2 and MET leads to the involvement of protein tyrosine phosphatase-1B (PTP1B) and the separation of adaptor proteins (such as Gab1, Grb2, Src, and PI3K), which inhibits MET dephosphorylation and enhances Src homology-2 domain-containing protein tyrosine phosphatase-2 (SHP2) phosphorylation (78). This shields RIG-I from degradation by SHP2/c-Cbl, enhancing IFN generation, and suppressing the replication of lymphocytic choriomeningitis virus (LCMV) (41), which implies that LECT2’s impact on metabolism and tumors arises through affecting MET and PTP1B (79).

Furthermore, MET can regulate LECT2 expression by activating β-catenin in turn (80). In mouse liver, LECT2 is directly targeted by Wnt/β-catenin, triggering LECT2 expression exclusively during β-catenin-dependent hepatocyte proliferation (28). However, LECT2 expression is not up-regulated in some HCC specimens containing β-catenin-activating mutations, indicating that Wnt/β-catenin is not the only pathway that regulates LECT2 expression in liver cancer. LECT2 can also interconnect with iNKT cells, which blocks β-catenin-induced inflammation and influencing liver tumor invasion in turn (51). Other studies have shown that LECT2’s tumor inhibition can also be achieved by inhibiting HCC glycolysis (81).

From the above studies, we can conclude that LECT2 may control HCC by three mechanisms: endothelial VEGF receptor signaling, MET signaling, and Wnt/β-catenin pathway. LECT2 may be a promising candidate for cancer therapy.

#### 4.3.3 Association of LECT2 with amyloidosis

ALECT2, a form of renal amyloidosis, is ranked as the third most prevalent type and was initially described by Benson et al.in 2008 (30). LECT2, a subunit protein in amyloid fibrils, has been identified as a protein capable of inducing systemic amyloidosis in cases of nephrotic syndrome and azotemia (31). This form of amyloidosis is common, accounting for 3 to 10 percent of amyloidosis reported abroad (82). Amyloidogenic LECT2 (ALECT2) forms clumps in various organs such as the lungs, spleen, adrenal glands, small intestine, gallbladder, ovaries, and uterus, but not in the heart or brain (83–86). Frequently diagnosed through clinical observation, chronic kidney failure is commonly accompanied by a mild presence of particles in the urine, with or without protein in the urine. Amyloid deposits in glomeruli, renal vessels, and stroma are common features in individuals suffering from nephrotic syndrome and renal failure (32, 87, 88).

LECT2-derived amyloidosis is ethnicity-related with a high prevalence in Hispanics (44, 89, 90). Excessive production of LECT2 leading to ALECT2 deposition is a prevalent factor in hepatic amyloidosis cases in the United States (44). Improved understanding and identification methods of this form of amyloidosis have led to a small amount of detection of ALECT2-induced amyloidosis in patients from Canadian Aboriginal northern British Columbia, Pakistan, Syria, and China who have chronic kidney disease (91–94). The implementation of laser microdissection and mass spectrometry (LMD/MS) has offered significant assistance in identifying and categorizing amyloidosis (87, 95). Analysis of LECT2 gene sequence in peripheral blood samples can serve as a predictive tool for LECT2 amyloidosis. In cases where all AL and AA markers yield negative results, further diagnostic measures should be taken to determine appropriate therapeutic interventions (96). The principle of non-maleficence should be prioritized, with cautious selection of potentially toxic treatments unless dealing with immunoglobulin light chain amyloidosis (46, 97–99). However, it is worth noting that ALECT2 remains inadequately investigated to date, leaving its pathogenesis still unclear.

### 4.4 Prospects for future treatments

There are no drugs on the market based on LECT2 as a therapeutic target. In the published study, there is only one drug development project for the LECT2 target by Alnylam Pharmaceuticals, Inc. The drug inhibits the expression of the LECT2 gene through RNA interference to treat amyloidosis. An existing patent provides a novel method for the treatment or prevention of tumors characterized by excessive activity of the Met receptor. Phosphorylation of MET in HCC cells is reduced by administration of agents that can increase the level or biological activity of the active fragment of the LECT2 protein in HCC cells. This method not only inhibits the proliferation, migration and invasiveness of HCC cells, but even includes human lung cancer cells, breast cancer cells, gastric cancer cells, ovarian cancer cells, hypopharyngeal cancer cells, colon cancer cells and glioma cells (100, 101). Gene therapy approaches targeting the LECT2 gene are also being explored. The DNA encoding the human LECT2 protein is transferred to cells for gene therapy by retroviral vectors or non-viral techniques such as receptor-mediated targeted DNA transfer, the use of ligand-DNA conjugates or adenovirus-ligand-DNA conjugates, lipid membrane fusion, or direct microinjection (102). LECT2 plays an important role in inflammation, immune response and tumor development, so it has the potential to be a target for future therapies. But more research is needed to verify its effectiveness and safety.

### 4.5 Advantages and shortcomings

There are multiple advantages to this research. The study utilized the Web of Science Core Collection Database, a comprehensive academic literature index database that includes reputable journals from multiple disciplines and is updated daily. As a literature search tool, it can ensure the credibility and high quality of bibliometric analysis data. Second, we used three bibliometric tools (Microsoft Office Excel 2021, VOSviewer, and CiteSpace) for visual analysis of bibliometrics. This method can reduce the distortion and bias caused by subjective information filtering. Finally, compared with the traditional review, the visual analysis and review of LECT2-related research in this study can more intuitively and comprehensively show the hot spots and frontiers in this field.

It’s worth noting that some limitations do exist in this study. Firstly, publications in non-SCI journals or other databases may not be included in the statistics. In addition, our study solely reflected the citation frequency or publication count to gauge the importance of a paper, which might not fully account for other factors.

## 5 Conclusion

A thorough bibliometric analysis was carried out in this research, examining the current literature on LECT2, including an assessment of publication dates, countries, organizations, authors, and publications. An in-depth examination of trends, research hotspots, and frontiers was also performed. The analysis results show that the current research has made a breakthrough thanks to the cooperation between countries and institutions, and more scholars have participated in LECT2 research. Research has shown that LECT2 holds significant potential for the treatment of immune diseases, liver fibrosis, liver cancer, amyloidosis, and other illnesses. However, the findings are preliminary and need more exploration for a clearer understanding.

## Data availability statement

Publicly available datasets were analyzed in this study. This data can be found here: Web of science.

## Author contributions

WL: Writing – original draft, Visualization. QW: Writing – original draft, Visualization. JY: Writing – original draft. YY: Writing – original draft. LX: Writing – original draft. CJ: Writing – review & editing. XL: Writing – review & editing, Funding acquisition. LZ: Writing – review & editing, Supervision, Project administration, Funding acquisition.

## Supplementary material

Supplementary File 1 Contains a table (Word) of the top 10 references that are frequently cited together in studies on LECT2 research and two images (TIF). One of the images shows the top 25 keywords with the strongest citation bursts. Another image is timeline viewer related to LECT2.
