# Supplementary figures and images for: Global research trends and hotspots for leukocyte cell-derived chemotaxin-2 from the past to 2023: a combined bibliometric review
Source: Front Immunol. 2024 May 31;15:1413466. doi: 10.3389/fimmu.2024.1413466 (PMC11176436; doi:10.3389/fimmu.2024.1413466)

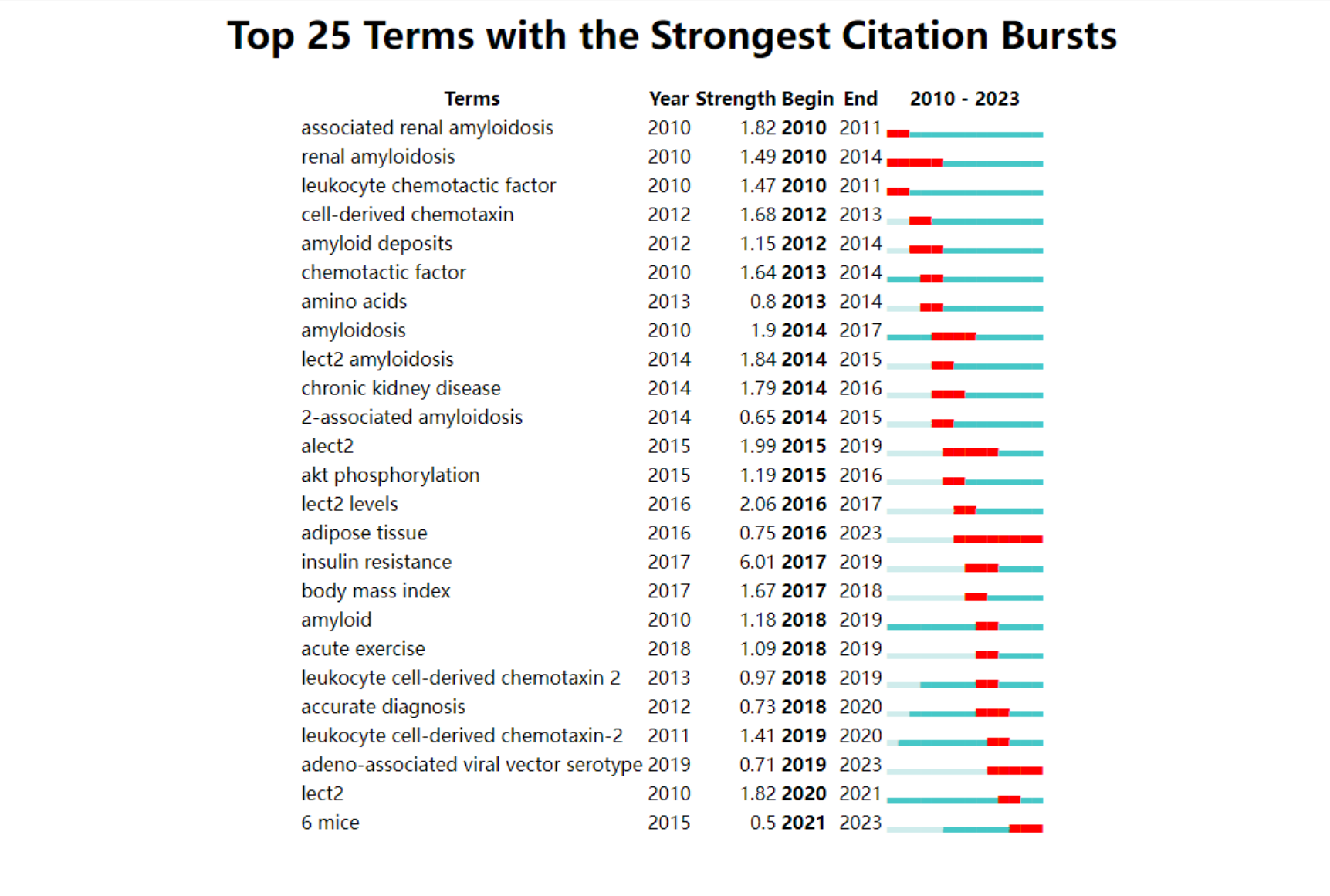

Supplement: Supplementary file 1 [file Image_1.tif]

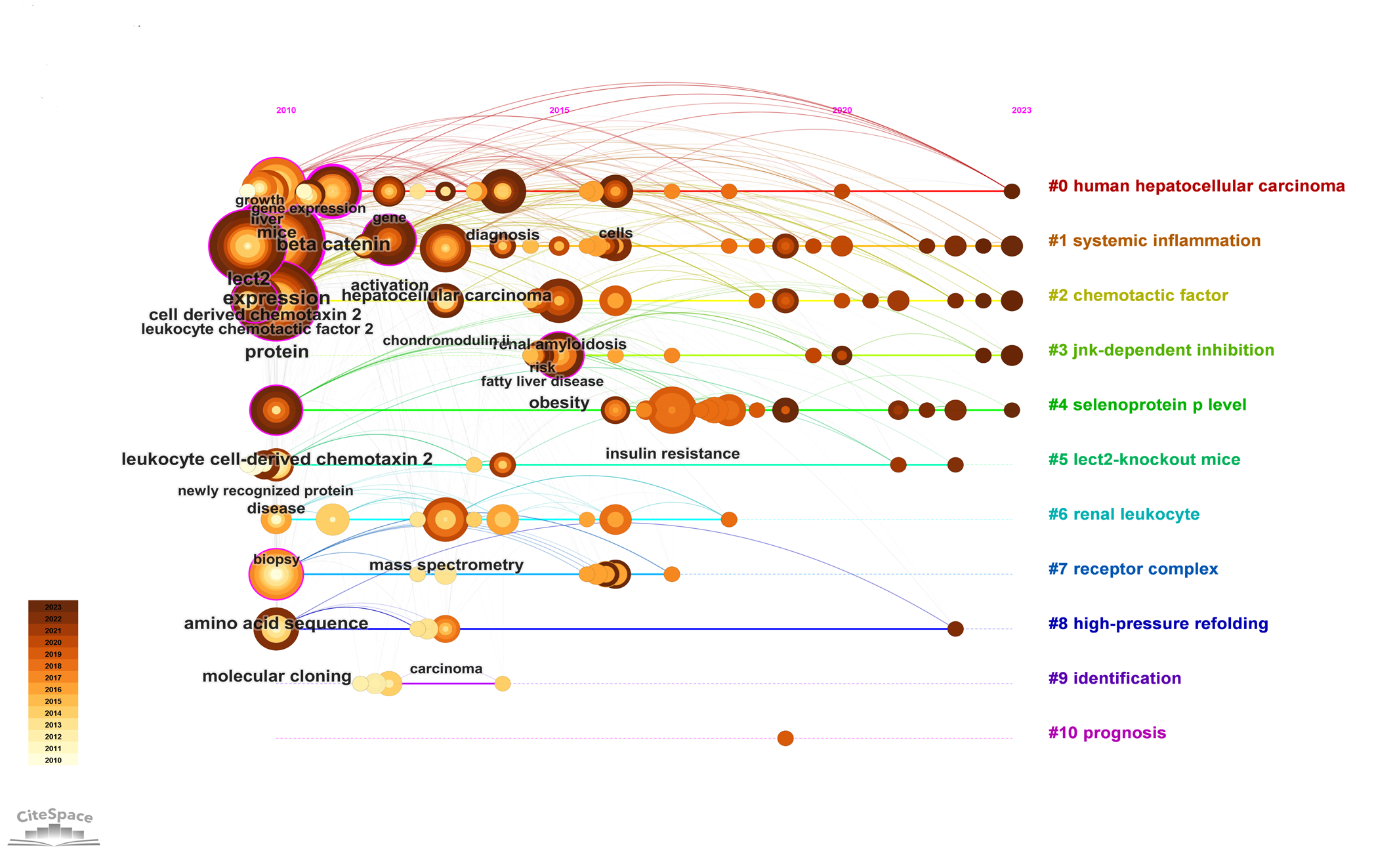

Supplement: Supplementary file 2 [file Image_2.tif]
