# Supplementary material for: Global research trends and hotspots for leukocyte cell-derived chemotaxin-2 from the past to 2023: a combined bibliometric review
Source: Front Immunol. 2024 May 31;15:1413466. doi: 10.3389/fimmu.2024.1413466 (PMC11176436; doi:10.3389/fimmu.2024.1413466)
Supplement: Supplementary File 1 — Contains a table (Word) of the top 10 references that are frequently cited together in studies on LECT2 research and two images (TIF). One of the images shows the top 25 keywords with the strongest citation bursts. Another image is timeline viewer related to LECT2. [file Table_1.docx]

**Supplementary Table 1.** Top 10 co-cited references on research of LECT2

| Rank | Title | Frequency | | Centrality | Journal | Author | Year |
| --- | --- | --- | --- | --- | --- | --- | --- |
| **1** | **LECT2, a Ligand for Tie1, Plays a Crucial Role in Liver Fibrogenesis** | **25** | **0.07** | | **CELL** | **Xu M** | **2019** |
| **2** | **LECT2 functions as a hepatokine that links obesity to skeletal muscle insulin resistance** | **24** | **0.13** | | **DIABETES** | **Lan F** | **2014** |
| **3** | **Prevalence and morphology of leukocyte chemotactic factor 2-associated amyloid in renal biopsies** | **17** | **0.1** | | **KIDNEY INT** | **Larsen CP** | **2010** |
| **4** | **LECT2 protects mice against bacterial sepsis by activating macrophages via the CD209a receptor** | **17** | **0.07** | | **J EXP MED** | **Lu XJ** | **2013** |
| **5** | **Leukocyte cell-derived chemotaxin 2 (LECT2)-associated amyloidosis is a frequent cause of hepatic amyloidosis in the United States** | **15** | **0.05** | | **BLOOD** | **Mereuta OM** | **2014** |
| **6** | **Increased serum leukocyte cell-derived chemotaxin 2 (LECT2) levels in obesity and fatty liver** | **15** | **0.11** | | **BIOSCI TRENDS** | **Okumura A** | **2013** |
| **7** | **LECT2 promotes inflammation and insulin resistance in adipocytes via P38 pathways** | **14** | **0.04** | | **J MOL ENDOCRINOL** | **Jung TW** | **2018** |
| **8** | **Lect2 Controls Inflammatory Monocytes to Constrain the Growth and Progression of Hepatocellular Carcinoma** | **14** | **0.16** | | **HEPATOLOGY** | **L'Hermitte A** | **2019** |
| **9** | **Clinical, morphologic, and genetic features of renal leukocyte chemotactic factor 2 amyloidosis** | **14** | **0.04** | | **KIDNEY INT** | **Larsen CP** | **2014** |
| **10** | **The tumor suppressor function of LECT2 in human hepatocellular carcinoma makes it a potential therapeutic target** | **14** | **0.13** | | **CANCER GENE THER** | **Ong HT** | **2011** |
